# Mammalian peripheral nerve specific autoimmunity: proposed mechanisms and redefinition of molecular mimicry

**DOI:** 10.3389/fneur.2026.1968458

**Published:** 2026-09-24

**Authors:** Eroboghene E. Ubogu

**Affiliations:** Division of Neuromuscular Disease, Department of Neurology, University of Alabama at Birmingham, Birmingham, AL, United States

**Keywords:** animal models, autoimmunity, blood-nerve barrier, effector memory CD4+ T cells, mechanism, molecular mimicry, peripheral nerves

## Abstract

Molecular mimicry is considered essential for peripheral nerve-specific autoimmunity: a process by which antibodies, systemically generated primarily as a consequence of infection with bacteria or viruses possessing epitopes that are similarly expressed by peripheral axon complex gangliosides or peripheral nerve myelin proteins, are hypothesized to induce peripheral nerve injury in genetically susceptible individuals. Human *in vitro* studies have proposed that recently activated antigen-specific effector memory CD4+ T-cells initiate tissue specific autoimmunity by T-cell receptor-driven transmigration following local microvasculature activation. However, how do these rare T-cells find their way from secondary lymphoid organs where they are generated to restrictively migrate to peripheral nerves and nerve roots to initiate an autoimmune response? Using severe murine experimental autoimmune neuritis as an animal model of peripheral nerve-specific autoimmunity, we elucidated that Major Histocompatibility Complex (MHC) class II expression on endoneurial endothelial cells is essential for disease onset using conditional knockout transgenic mice. This implies that specific BNB antigen-expressing MHC Class II molecules could serve as a directional cue for antigen-specific effector memory CD4+ T-cell transmigration: an active, molecular-dependent, coordinated multi-step mechanistic paradigm, to initiate tissue-specific autoimmunity and drive subsequent monocyte and B-cell infiltration, resulting in peripheral nerve demyelination and axonal degeneration. Based on these initial observations, I propose to redefine molecular mimicry as follows: The process by which recently activated antigen-specific effector memory CD4+ T-cells engage with MHC class II-expressing microvascular endothelial cells that present the same processed antigen peptide sequences during immunosurveillance to trigger their transmigration and initiate tissue-specific autoimmunity.

## Introduction

Autoimmune disorders affect ~3%–5% of the world’s population (1). Incidence and prevalence continue to rise (1, 2). Annual direct costs in the United States of America are conservatively estimated at over $100 billion (2). Despite significant advances in the pathogenesis, diagnosis and treatment of autoimmune disorders, major knowledge gaps exist. Fewer advances have been made in peripheral nerve-specific autoimmune disorders like Guillain-Barré syndrome (GBS). This is a severe autoimmune disorder that causes varying degrees of muscle weakness, sensory loss, autonomic dysfunction and cardiopulmonary compromise (3). GBS is a disorder that affects adults in a ~ 1.3:1 male-to-female ratio irrespective of race, ethnicity and socioeconomic status, and there is increased prevalence with advancing age (4). Consistent with other autoimmune disorders, GBS prevalence is increasing worldwide (4). Unlike chronic inflammatory demyelinating polyradiculoneuropathy (CIDP) and vasculitic neuropathy that may occur with systemic autoimmune disorders, GBS is restricted to peripheral nerves and nerve roots, rendering this disorder ideal to understand how tissue-specific autoimmunity may occur in peripheral nerve and nerve roots.

Primary adaptive immunity is initiated by exogenous antigen presentation to naïve CD4+ T-cells by “professional” myeloid antigen-presenting cells (APCs), e.g., dendritic cells, macrophages and B-cells, residing in secondary lymphoid organs (5–8). CD4+ T-cells principally mediate surveillance through their T cell receptor (TCR) and are restricted to MHC class II molecules that present extracellular or phagocytosed peptides. Following systemic immune activation that may occur after infections, trauma, surgery and stress, naïve CD4+ T-cells undergo co-stimulation through selective receptors and cytokines, become highly activated resulting in a burst of proliferation and differentiation into multiple effector subsets that are designed to participate in various immune responses. Most effector CD4+ T-cells are short-lived, but a small fraction of them of form long-lived memory cells that persist long beyond pathogen clearance. This includes effector memory CD4+ T-cells that persist long after initial antigen clearance, and rapidly initiate secondary tissue responses, geared to quickly protect the organism from future tissue damage following re-exposure to the same antigen(s) or specific antigen reappearance (5–10).

Molecular mimicry is a hypothesized process by which infections, chemical agents and tissue injury (e.g., surgery) induce autoimmunity due to similarities between exogenous and endogenous peptides, resulting in self-reactive T- or B-cell activation in susceptible individuals (7). Patient age, sex, genetic and epigenetic factors, prior pathogen exposures, vaccination status, microbiome and other environmental exposures are proposed to influence molecular mimicry (6–8). Dysregulated central tolerance, non-specific bystander activation, persistent antigenic stimuli, epitope shift/ drift and downregulation in regulatory CD4+ T-cells are also proposed to contribute to autoimmune disease initiation, relapse and persistence (6, 11). Molecular mimicry is considered essential to GBS pathogenesis, with pathogenic antibodies generated against peripheral axon complex gangliosides or peripheral nerve myelin proteins or lipids following exposure to pathogens such as bacteria or viruses. These microorganisms endogenously express epitopes that share molecular similarities to peripheral nerve antigens, As a consequence, the immune responses generated to eradicate these microorganisms inadvertently cause peripheral nerve and nerve root demyelination and axonal injury (12–14). However, most individuals exposed to these pathogens do not develop GBS, so genetic susceptibility factors are cited to explain patient selectivity. It is important to recognize that definitive pathogenic antibodies responsible for the most common GBS variant worldwide, acute inflammatory demyelinating polyradiculoneuropathy (AIDP), remain elusive, as is the case for CIDP and its variants. MHC or Human Leukocyte Antigen (HLA) class II susceptibility risk genes have not been definitively identified in GBS, with several candidates proposed (15–18). Based on cytokine profiling, *in vitro* T cell screening, single-cell RNA sequencing and T cell receptor (TCR) sequencing, there is emerging evidence that AIDP-GBS is associated with autoreactive effector memory CD4+ T cells with a T-helper 1 (Th1)-like phenotype that target peripheral nerve myelin antigens, implying an essential pathogenic role of CD4+ T-cells in this autoimmune disorder (19–22).

Histopathological evaluation of AIDP-variant GBS patient biopsies supports hematogenous leukocyte trafficking into the endoneurium, with monocyte/ macrophage, CD4+ and CD8+ T-cell and B-cell/ plasma cell infiltration observed in association with demyelination and axonal degeneration (23–28). Importantly, electron dense intercellular contacts between endoneurial endothelial cells, indicative of tight junctions, are present during pathogenic leukocyte trafficking in AIDP, supporting the notion that leukocyte trafficking across microvessels is an active, molecular-driven, coordinated, sequential process (i.e., multistep paradigm of leukocyte trafficking) dependent on dynamic leukocyte-endothelial cell interactions *in vivo* as seen *in vitro* (27, 29–33), rather than passively due to a “leaky” blood-nerve barrier (BNB). Inflammatory leukocyte migration across microvessels is driven by specific selectins, chemokines and adhesion molecules on endothelial cells, and selectin counterligands, chemokine receptors, integrins and matrix metalloproteases expressed by leukocytes (28, 30, 32, 34, 35).

### Restricted trafficking of antigen-specific effector leukocytes to peripheral nerves

The initial directional cues for antigen-specific effector or autoreactive leukocyte trafficking into peripheral nerves during autoimmunity are unknown. How do recently activated effector memory CD4+ T-cells generated in secondary lymphoid organs, efficiently migrate to peripheral nerves and nerve roots alone to induce demyelination and axonal degeneration in susceptive individuals as part of aberrant adaptive immune response without causing injury to other tissues or organs? Based on published human *in vitro* and *ex vivo* studies, effector memory CD4+ T-cells (characterized as CD4+ CD25− CD44+ CD45RA− CD45RO+ CD62L− CD127+) are proposed to migrate into tissues following local microvasculature activation by inflammatory cytokines and pathogen-associated or damage-associated molecular patterns (5, 9). These studies, using cultured dermal, lung or brain microvascular endothelial cell layers demonstrated that initial antigen-specific CD4+ T-cell transmigration under static or flow conditions was dependent on endothelial cell MHC Class II antigen presentation (36–39). The formation of TCR-driven transendothelial protrusions by effector memory CD4+ T-cells was shown as the initial step for rapid paracellular migration across microvascular endothelial cell layers under flow conditions, in contrast to arrested adhesion on human umbilical vein macrovascular endothelial cell layers *in vitro* (36, 39). Selective chemokine and cell adhesion molecules were shown to attract, activate and facilitate antigen-specific CD4+ T-cell transmigration *in vitro* (36, 40, 41). Endothelial cell MHC Class II antigen presentation was also shown to induce effector memory CD4+ T-cell activation and proliferation *in vitro*, with increased evidence supporting a direct role in antigen-specific T-cell transmigration and allograft transplant rejection (37, 38, 42–44). Importantly, only highly activated neuritogenic myelin peripheral nerve peptide P2_55-78_-reactive T-cells, and not non-specific T-cells, were able to cross the BNB in a Lewis rat adoptive transfer experimental autoimmune neuritis (EAN) model of GBS by an undetermined mechanism (45), suggesting that endoneurial endothelial cell-antigen specific T cell interactions at the BNB were necessary for peripheral specific autoimmunity *in vivo*.

We showed that several HLA-D transcripts were expressed by human endoneurial endothelial cells *in situ* based on laser capture microdissection of endoneurial microvessels from histologically normal adult sural nerves that were conserved *in vitro* by primary human endoneurial endothelial cells (46). We also observed that human endoneurial endothelial cells expressed the constitutive transcript repertoire of non-professional antigen presenting cells *in situ* and *in vitro* (46). These observations suggest that human endoneurial endothelial cells may serve as facultative APCs during physiologic and pathogenic immune responses. However, *in vitro* transmigration studies do not fully recapitulate what may occur in microvessels *in vivo*. Increased MHC Class II (HLA-D) expression had been shown on multiple cell types: monocytes/ macrophages, Schwann cells, infiltrating T-cells, perineurial cells and BNB-forming endoneurial endothelial cells in AIDP-variant GBS and CIDP patient nerve biopsies compared to non-inflammatory neuropathy controls (47–53). However, nerve biopsies were typically not performed at disease onset in GBS patients, so these observational studies do not clarify whether peripheral nerve MHC class II expression is necessary for GBS pathogenesis, and if so, expression on which cell type(s) is/are pathogenically relevant.

In order to determine whether endothelial MHC Class II expression is necessary for tissue-specific autoimmunity *in vivo*, we studied the severe murine experimental autoimmune neuritis (sm-EAN) model of AIDP-variant GBS in female SJL mice as an example of peripheral nerve-specific autoimmunity. This is a disorder induced by inoculation of bovine peripheral nerve myelin emulsified in complete Freund adjuvant with pertussis toxin and recombinant mouse interleukin-12 administrated as co-adjuvants to drive an acute, self-limiting CD4+ T helper 1-mediated inflammatory demyelination with axonal degeneration. 100% disease development and maximum disease severity occur in 8–12 week old female mice, with disease onset 7–10 days after induction (27, 54–56). We hypothesized that recently activated circulating effector memory CD4+ T-cells produced in secondary lymphoid organs after autoantigen exposure, bind to specific antigen-expressing MHC class II molecules on peripheral nerve/nerve root endoneurial endothelial cells during normal immunosurveillance; however, recognition of peripheral nerve antigens that are the same as those that triggered their initial differentiation and proliferation initiates local transmigration and initiation of tissue-specific autoimmunity. This provides a plausible explanation why autoreactive peripheral neuritogenic CD4+ T-cells would migrate across the BNB, but not non-specific activated CD4+ T-cells in adoptive transfer Lewis rat EAN (45).

### Direct evidence of functional role of endothelial MHC class II expression in peripheral nerve specific autoimmunity *in vivo*

Mouse MHC class II molecules consists of a 33 kDa α-chain covalently linked to a 28 KDa β-chain. Mice possess 2 α-chains, H2-Aa and H2-Ea, homologous to human HLA-DQA1 and HLA-DRA molecules, respectively. Classical (H2-Ab1 and H2-Eb1) and non-classical (H2-Ob [H2-Ab2] and H2-Eb2) β-chains can bind to the highly conserved α-chains to form effective antigen presentation MHC class II complexes (57). Mouse strains commonly used to model human autoimmune disorders (including AIDP-variant GBS) are H2-Ea germline knockouts, implying that experimental autoimmunity in these mouse strains is H2-Aa-dependent. Multiple cell types express MHC class II and may serve as professional, semi-professional or facultative antigen presenting cells under normal physiologic or pathophysiologic conditions. In order to study cell- and time-dependent adaptive immune responses relevant to tissue-specific autoimmunity *in vivo*, we developed a novel MHC class II A-alpha chain conditional knockout (*H2-Aa*^flox/flox^) SJL mouse strain (SJL.B6-*H2-Aa^tm1c(KOMP)Wtsi^*/UbeeMmmh; RRID: MMRRC_071292-MU). These mice were developed from our initial C57BL/6-*H2-Aa^b-tm1c(KOMP)Wtsi^*/UbeeMmmh; RRID: MMRRC_046053-MU mouse strain deposited with the Mutant Mouse Research & Resource Center (MMRRC) by speed congenics. Of note. EAN is a mild, variable-onset disorder in C57BL/6 mice but severe and stereotypical in female SJL mice (53, 58).

It is also important to recognize that functional and phenotypic differences exist between microvascular and macrovascular endothelial cells in different tissues, organs and species (59–63). Due to limitations in mouse strains developed for microvascular endothelial cell germline or conditional gene knockout (64), and a desire to study time-dependent endothelial-specific gene knockout in adult mice, we also developed a Tamoxifen-inducible hemizygous von Willebrand factor Cre recombinase (vWF-iCre/+) transgenic SJL mouse strain with a human vWF genomic DNA fragment containing the 734 base pair microvascular endothelial cell-specific promoter sequence (65, 66) to drive Cre recombinase, fused to a mutant estrogen ligand-binding domain that requires Tamoxifen for activity, followed by a polyadenylation signal (SJL Cg-Tg(VWF-Cre/ERT2)C1014/UbeeMmmh; RRID: MMRRC_071293-MU) (67). Detailed characterization of these mouse strains have been published (53, 67).

To study microvascular endothelial cell-specific MHC class II gene knockout in peripheral nerve tissue-specific autoimmunity, we crossbred these SJL mouse strains and generated a Tamoxifen-inducible microvascular endothelial cell-specific MHC class II conditional knockout SJL mouse strain (*H2-Aa*^flox/flox^; vWF-iCre/+). Our construct selectively excised exons 2, 3, 4 and the 5′ portion of exon 5 in Cre recombinase-expressing cells, generating a nonfunctional H2-Aa protein (53). After maximal Tamoxifen-induced gene recombination which deletes MHC Class II from microvascular endothelial cells only, we induced sm-EAN in 11–12 week old female SJL mice via subcutaneous injections of bovine peripheral nerve myelin (BPNM) emulsified in Complete Freund Adjuvant (CFA), with Pertussis toxin and recombinant mouse interleukin (IL)-12 serving as co-adjuvants with appropriate controls, as previously published (27, 54–56, 68). We performed daily weights and neurobehavioral assessments using our published neuromuscular severity scale (NMSS), and performed motor nerve electrophysiology studies and harvested sciatic nerves, inguinal lymph nodes and spleens on day 28 post-induction (during peak severity), as previously published (27, 55, 56, 69) or publically archived (53).

Tamoxifen-treated female *H2-Aa*^flox/flox^; vWF-iCre/+ SJL mice with microvascular endothelial cell *H2-Aa* deletion maintained normal body weights and did not develop sm-EAN based on NMSS, exhibiting normal sciatic and dorsal caudal tail nerve motor electrophysiology and no histological evidence of sciatic or dorsal caudal tail nerve inflammation, demyelination or axonal loss for up to 30 days after disease induction (53). We also observed leukocytes in the connective tissue external to the sciatic and dorsal caudal tail nerves, implying these leukocytes had infiltrative capacity, but failed to transmigrate to peripheral nerves (53). Tamoxifen-treated female *H2-Aa*^flox/flox^; vWF-iCre/+ SJL mice also had normal bone marrow, lymph node and spleen MHC Class II expression by indirect fluorescent immunohistochemistry (53). This study provided the initial proof that microvascular endothelial cell MHC class II expression was a prerequisite for murine peripheral nerve specific autoimmunity, suggesting that MHC class II provides the directional cue for antigen-specific effector memory CD4+ T-cell transmigration across the BNB during autoimmunity. When developing our SJL mouse strain, we observed sequence differences in *H2-Aa* exon 2 between SJL and C57BL/6 mice 5′ to the transgene insertion site (53) that could explain differential susceptibility to EAN (58).

## Discussion

Our initial studies show that microvascular endothelial cell-specific H2-Aa knockout prevented acute autoimmune demyelinating neuritis in female adult SJL mice, implying that endothelial cell MHC Class II expression at the BNB is necessary for antigen-specific peripheral nerve-restricted autoimmunity. This supports human *in vitro* data that demonstrated vascular endothelial cell capacity to promote antigen presentation, effector memory T-cell proliferation, and antigen-specific CD4+ T-cell migration (5, 36–38, 43), and the restricted migration of highly activated peripheral neuritogenic T-cells across the BNB in adoptive transfer Lewis rat EAN (45). Importantly, leukocyte H2-A expression was not unexpectedly altered in Tamoxifen-treated H2-Aa^flox/flox^; vWF-iCre/+ SJL mouse bone marrow or secondary lymphoid organs to suggest dysregulated professional APC function required to initiate adaptive immune responses to exogenous antigens following naïve T-cell activation. Despite the limitations of murine models of human autoimmune disease, these observations provide pathogenic insights into the initiation of human AIDP.

SJL mouse adaptive immune responses require H2-Aa, as these mice are H2-Ea null and redundancy exists for β-chain molecules.^57^ H2-Aa (with human homolog HLA-DQA1) is highly conserved between species, suggesting an essential role in adaptive immunity. Polymorphisms (e.g., at exon 2) could explain differential susceptibility to sm-EAN between SJL and C57BL/6 mice. Although conclusive HLA class II susceptibility risk genes have not been identified in GBS (15–18). HLA-DQA1 is a plausible target for deep sequencing in worldwide AIDP-GBS patient cohorts. Based on these initial observations, I propose to redefine molecular mimicry as follows: The process by which recently activated antigen-specific effector memory CD4+ T-cells engage with MHC class II-expressing microvascular endothelial cells that present the same processed antigen peptide sequences to trigger their transmigration and initiate tissue-specific autoimmunity.

Guided by prior published *in vitro* studies using flow-dependent human dermal and lung microvascular and endoneurial endothelial cell transmigration models, data from the human BNB transcriptome and our initial single cell RNA sequencing of untreated GBS (AIDP variant) patient leukocytes (5, 22, 36, 37, 41, 42, 46, 70), I propose that recently activated effector memory CD4+ T helper 1 cells roll on microvascular endothelial cell surfaces via P-selectin-P-selectin glyprotein-1 interactions and undergo haptotaxis due to expression of chemokine receptor CX3CR1 that binds to constitutionally expressed luminal CX3CL1 on endoneurial microvessels. Antigen presentation occurs to these effector memory CD4 Th1 TCR via endothelium MHC Class II (alpha chain dependent). If there is a mismatch, these effector memory cells disengage and move on (as observed at the human BNB *in vitro*) (71, 72). If there is a match, co-stimulation occurs (e.g., via CD40L-CD40, ICOS-ICOSL interactions) which further activates the effector memory CD4+ T cell converting their inactive integrins such as CD11b and Cd49d to active integrins via conformational change. These activated integrins bind to constitutively expressed adhesion molecules such as ICAM-1 and VCAM-1 resulting in firm adhesion which leads to paracellular diapedesis via homophilic interactions between T-cell and endothelial cell CD99 (Figure 1). The proposed molecular determinants and signaling mechanisms required for antigen-specific effector memory CD4+ T-cell engagement with endoneurial endothelial cells via MHC class II as a prerequisite for pathogenic leukocyte transmigration and acute demyelinating neuritis in sm-EAN, requires mechanistic validation that is only feasible using representative animal models as the initiation of an autoimmune response occurs pre-symptomatically in clinically affected patients. These signaling pathways may have potential relevance to other immune-mediated polyneuropathies, such as CIDP.

**Figure 1:**
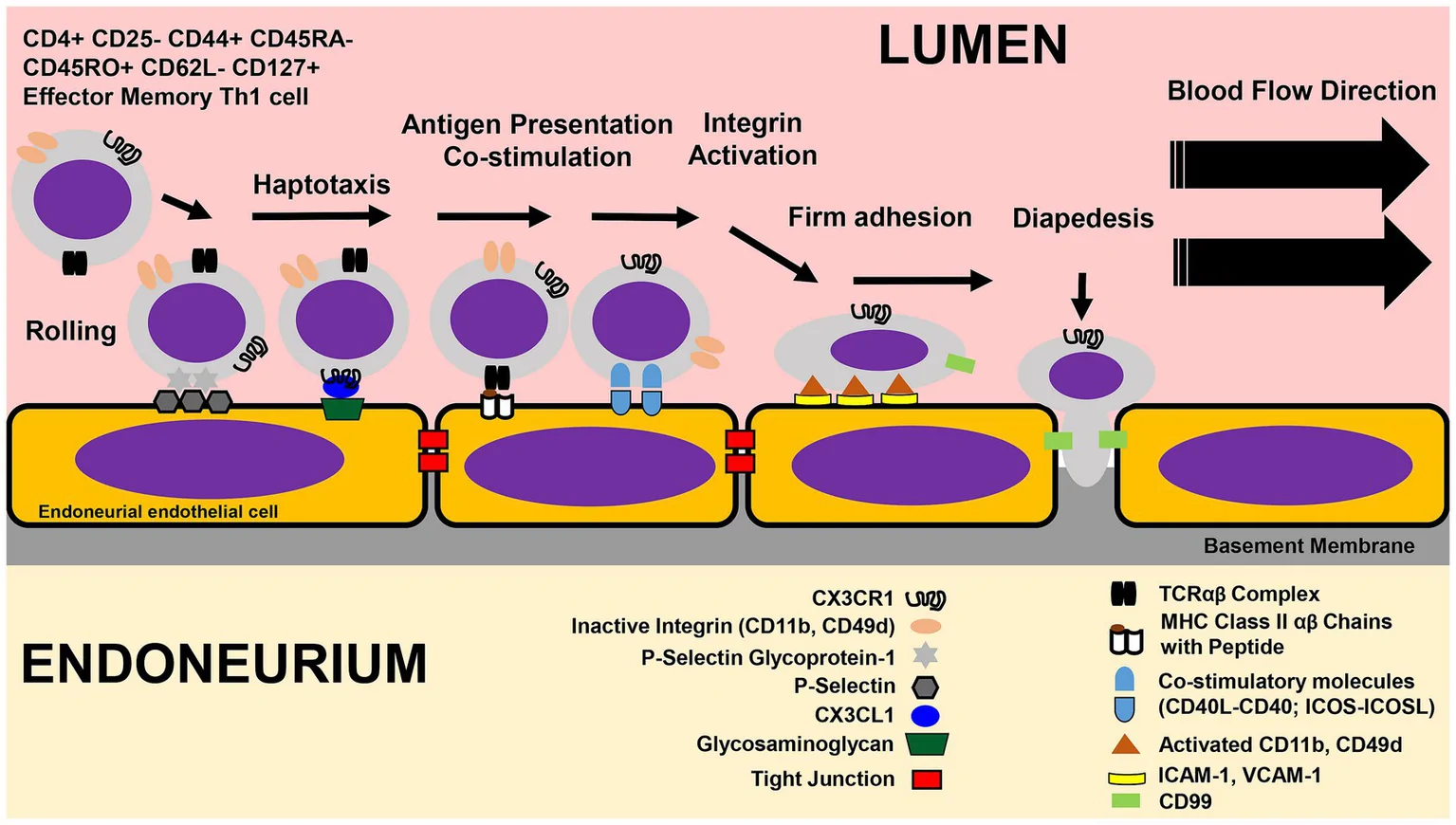
Induction of peripheral nerve tissue-specific autoimmunity. This figure depicts the hypothesized cascade of events that triggers restricted autoimmune responses in peripheral nerves, guided by published data on human TCR-dependent effector memory CD4+ trafficking *in vitro* and the molecular composition of BNB-forming human endoneurial endothelial cells *in situ*. Recently activated antigen-specific effector memory CD4+ T helper 1 cells derived from secondary lymphoid organs (e.g., from local lymph nodes) enter into the bloodstream to facilitate immunosurveillance. These T-cells roll on endoneurial endothelial cell surfaces, e.g., via P-selectin-P-selectin glyprotein-1 interactions and undergo haptotaxis in response to constitutively expressed chemokines, e.g., CX3CL1, expressed on the luminal surface of endoneurial endothelial cells (membrane bound or bound by glycosaminoglycans) using chemokine receptor CX3CR1. MHC class II-mediated antigen presentation by endoneurial endothelial cells occurs to the effector memory CD4 Th1 TCR. Following antigen/ epitope recognition, co-stimulation takes place (e.g., via CD40L-CD40, ICOS-ICOSL interactions) which further activates the effector memory CD4+ Th1 cell converting their inactive integrins such as CD11b and Cd49d to active integrins via conformational change. These activated integrins bind to constitutively expressed adhesion molecules such as ICAM-1 and VCAM-1 resulting in firm adhesion which leads to paracellular diapedesis via homophilic interactions between T-cell and endoneurial endothelial cell (e.g. CD99) or other homophilic or heterophilic molecular interactions that support transmigration. These effector memory CD4+ Th1 cells can migrate freely within the endoneurium, release cytokines and activate endogenous macrophages to initiate an autoimmune response.

Conditional H2-Aa knockout mouse strains provide essential tools to study antigen presentation by different cells during tissue- or organ-specific adaptive immune responses, as Tamoxifen-induced Cre recombination effectively facilitates timed gene deletion to the phase of the autoimmune disease model (i.e., initiation, progression, recovery etc.) (73). Such studies would aid ascertain the role of MHC Class II in gastrointestinal and respiratory tract epithelial mucosal immunity *in vivo* (74–76), and determine whether blood–brain barrier microvascular endothelial cell MHC class II expression is a prerequisite for the initiation or restricted to amplification of autoimmune neuroinflammation *in vivo* (77). Tissue or organ grafts from mice with microvascular endothelial cell-specific MHC class II knockout could provide insights into allograft and xenograft rejection *in vivo*. Studies using living mice with intact immune systems are advantageous over human *in vitro* and *ex vivo* studies to better understand tissue-specific autoimmunity, provided such studies are guided by well-characterized patient data. Improved understanding of molecular mimicry, tissue-specific autoimmunity and transplant rejection obtained from conditional MHC class II knockout mouse studies should advance knowledge and guide targeted immune drug development strategies beyond peripheral nerves.

## Data Availability

The original contributions presented in the study are included in the article/supplementary material, further inquiries can be directed to the corresponding author.
